# Unique Reporter-Based Sensor Platforms to Monitor Signalling in Cells

**DOI:** 10.1371/journal.pone.0050521

**Published:** 2012-11-29

**Authors:** Meesbah Jiwaji, Rónán Daly, Abdullah Gibriel, Gráinne Barkess, Pauline McLean, Jingli Yang, Kshama Pansare, Sarah Cumming, Alisha McLauchlan, Piotr J. Kamola, Musab S. Bhutta, Adam G. West, Katherine L. West, Walter Kolch, Mark A. Girolami, Andrew R. Pitt

**Affiliations:** 1 Institute of Molecular, Cell and Systems Biology, College of Medical, Veterinary and Life Sciences, University of Glasgow, Glasgow, United Kingdom; 2 School of Computing Science, University of Glasgow, Glasgow, United Kingdom; 3 Institute of Cancer Sciences, College of Medical, Veterinary and Life Sciences, University of Glasgow, Glasgow, United Kingdom; 4 Systems Biology Ireland and the Conway Institute, University College Dublin, Dublin, Ireland; 5 Department of Statistical Science, University College London, London, United Kingdom; 6 School of Life and Health Science, Aston University, Birmingham, United Kingdom; H.Lee Moffitt Cancer Center & Research Institute, United States of America

## Abstract

**Introduction:**

In recent years much progress has been made in the development of tools for systems biology to study the levels of mRNA and protein, and their interactions within cells. However, few multiplexed methodologies are available to study cell signalling directly at the transcription factor level.

**Methods:**

Here we describe a sensitive, plasmid-based RNA reporter methodology to study transcription factor activation in mammalian cells, and apply this technology to profiling 60 transcription factors in parallel. The methodology uses two robust and easily accessible detection platforms; quantitative real-time PCR for quantitative analysis and DNA microarrays for parallel, higher throughput analysis.

**Findings:**

We test the specificity of the detection platforms with ten inducers and independently validate the transcription factor activation.

**Conclusions:**

We report a methodology for the multiplexed study of transcription factor activation in mammalian cells that is direct and not theoretically limited by the number of available reporters.

## Introduction

Analysis of the human genome has assigned function to almost 60% of the DNA sequence, based on known function or predicted similarity to known proteins. Of these, some 1850 (6%) are predicted to be transcription factors (TFs) [1], crucial components of cellular regulatory networks that dictate complex cellular phenotypic programs [2].

In eukaryotes, gene transcription is usually regulated by multiple TFs [3]–[4], and individual TFs contribute to the combinatorial control of the activation of a number of different genes [3], [5]–[7]. The large number of potentially interacting TFs and multiple target genes makes the gene-level experimental identification of specific TF activity in a cell technically difficult and time consuming. This has necesitated the development of bioinformatics-based approaches which predict specific TF interaction inferred from global gene expression data and putative TF binding sequences present in regulatory regions [8]–[11]. These well-established gene expression profiles and validated TF activities are used to train the model algorithms; however, many of the TFs predicted by such analyzes to play roles in specific tissues have not yet been confirmed experimentally. The direct analysis of the biochemical activities of the TFs themselves would, therefore, be of great value to biochemical and systems biological research.

Only a few studies have described experimental methods to systematically detect TF activation in response to intracellular signalling [7], [12]–[13]. Qiao *et al*. [7] reported an array-based approach for the analysis of the binding activities of TFs. Here, proteins bound to specific labelled DNA binding sequences were separated on an agarose gel and the TF-bound DNA was purified from the gel and analyzed using DNA arrays. This method is dependent on the *in vitro* binding of the TFs to the respective DNA binding sequences, the interaction being strong enough to withstand gel electrophoresis, and sufficient resolving power in the gel. Romanov *et al*. [12] and Botvinnik *et al.* [13] both described reporter based systems to study intracellular signalling. The Romanov system used homogenous reporters where TF binding sites (TFBS) were inserted upstream of reporters that differed in the position of a restriction enzyme site. Cellular mRNA transcripts were amplified and labelled, the products were digested and separated based on their length, before being detected and quantified. The Botvinnik system differed in the design of the reporters, which were in 4-letter ‘words’, and in this system the transcripts isolated from cells were amplified, labelled and then analyzed on an array. While these approaches have provided tantalizing evidence for the potential power of systematic technologies to analyze TFs, they also have weaknesses. They all require an enzymatic labelling step where efficiencies can vary and significant loss of product or signal can occur. Also, each system relies on a single detection method and thus there is no way to independently validate the data within the experiment.

**Figure 1:**
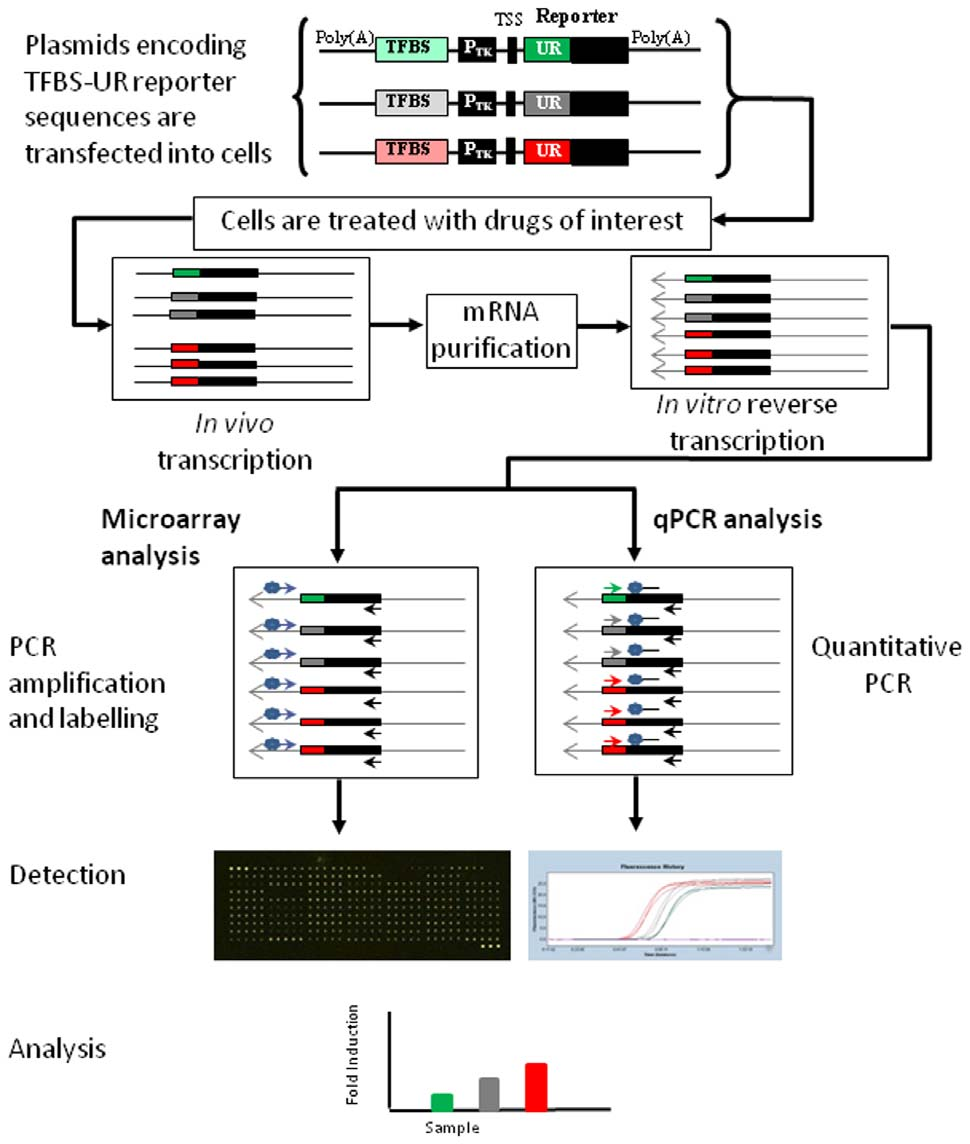
A schematic representation of the method. In each reporter plasmid, the transcription factor binding site (TFBS) and the thymidine kinase promoter (P_TK_) were present upstream of the transcriptional start site (TSS) and the unique DNA reporter (UR) sequence. The cassette was flanked by two poly(A) signals to prevent transcriptional interference due to the circular plasmid. Each TFBS was assigned a specific UR sequence to act as a signature for its corresponding TF activity. These plasmids were tranfected into cells and the cells treated with compounds of interest, mRNA was isolated, reverse transcribed and analyzed on two detection platforms. For microarray analysis, cDNA was amplified by PCR using a Cy3 or Cy5-labelled universal sense forward primer (Cy3/Cy5-AG_URF) in conjunction with a universal antisense reverse primer (prMJ264) to generate a mixture of 120 bp fluorescently labelled PCR amplicons that could be analyzed on DNA microarrays. For the qPCR reaction, a forward primer, specific for each UR, was used in combination with a universal FAM-labelled hydrolysis probe (prMJ245) and a universal reverse primer (prMJ264).

Here, we describe a reporter based system utilizing two detection platforms, based on quantitative real-time PCR (qPCR) and microarrays, for the measurement of TF activity in mammalian cells. These have the advantage that qPCR is sensitive and reproducible, has a large dynamic range and allows the simultaneous analysis of multiple samples, which makes it suitable for applications where cell numbers are limited [14]–[16]. Microarrays are sensitive [17]–[18] and relatively cheap making high throughput analysis of large experiments feasible [19]. Accurate quantification using microarrays remains a challenge. Therefore, we apply these methods as two independent platforms; microarrays as high throughput screens and qPCR for the accurate quantification of TF activity. With our current library of 60 TFs, and using ten different treatments, we show that our system is specific and sensitive, scalable, and allows the simultaneous detection of multiple TF activities.

**Figure 2:**
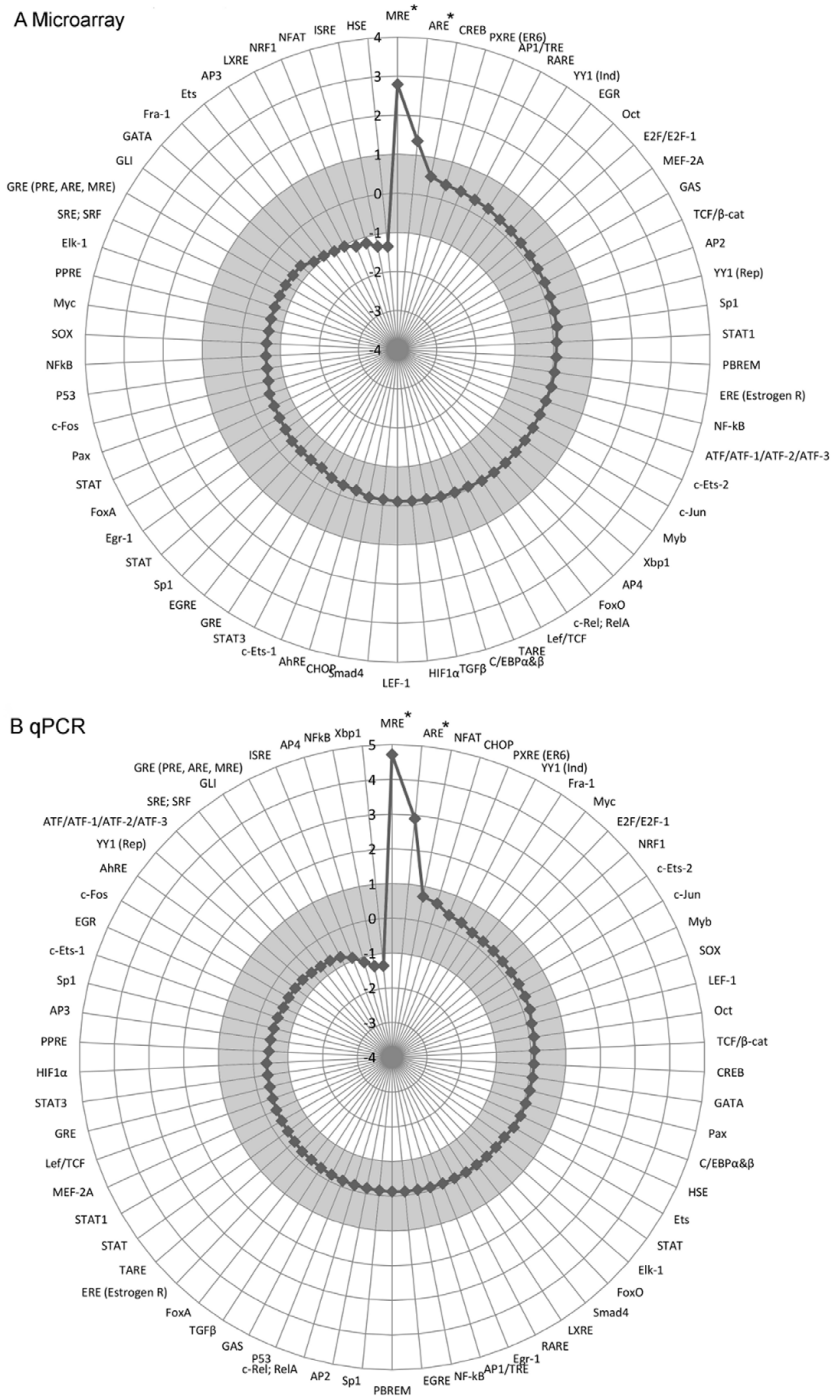
Analysis of induction in cadmium chloride-treated cells transfected with TFBS-UR plasmids. HEK293 cells transfected with a plasmid pool, that included the plasmids listed in Table S2 and pRL-SV40 and were subsequently treated with cadmium. (A) Microarray-based detection of TF derived activation of UR expression. (B) qPCR-based detection of TF-derived activation of UR expression. Values are presented as log2 treatments of the fold induction of the TFBS-directed UR expression after treatment with the inducer of interest. The grey bar represents treatment-independent changes in the system. TFBS marked with * represent treatment-dependent effects on the TF library. Numerical data is presented in Table S3. A statistical analysis of the qPCR assay data is shown in Figure 3.

## Methods

All quantification experiments contained four biological replicates and each experiment was repeated at least three times.

### Chemicals

All chemicals are from Sigma unless otherwise stated. Phosphodiesterase inhibitors were obtained from Calbiochem unless otherwise stated.

### General Culture Conditions

*Escherichia coli* DH5α cells were used for the construction, screening and propagation of plasmid constructs as described in Jiwaji *et al*. [20]. HEK293 cells (ATCC Number CRL-1573) were maintained in DMEM supplemented with 4 mM L-glutamine and 10% FBS at 37°C in an atmosphere that contained 5% CO_2_.

### Plasmid Description

DNA encoding a multiple cloning cassette and thymidine kinase promoter (P_TK_) was inserted between *Kpn* I and *Hind* III upstream of the firefly luciferase gene (*Fluc*) in pGL3 Basic (Promega) generating pMN2. DNA encoding TFBS sequences (Table S2) were inserted upstream of P_TK_. *Fluc* was then replaced with a unique DNA reporter sequence (UR) such that each TFBS was attached to a different UR (Table S2). The sequence of an example plasmid has been submitted to GenBank (Accession number GU217589).

**Table 1:** Activation of transcription factors by specific treatments on the qPCR platform.

Treatment	TFs	Log2 Fold Induction	p-value
Dexamethasone	GRE	3.8±0.6	1.0×10^−9^
	GRE (PRE/ARE/MRE)	2.9±0.4	1.0×10^−9^
	SRE/SRF	2.0±0.3	1.0×10^−9^
TPA	NF-κB	4.4±0.6	1.0×10^−9^
	c-Rel; RelA	3.9±0.4	1.0×10^−9^
	NF-κB	3.2±0.2	1.0×10^−9^
	c-Jun	3.2±0.7	1.0×10^−9^
	AP1/TRE	2.3±0.4	2.2×10^−5^
Forskolin	CREB	13.6±0.5	1.0×10^−9^
	ATF/1/2/3	12.8±0.1	1.0×10^−9^
	SRE/SRF	3.3±0.2	1.0×10^−9^
8-bromo-cAMP	CREB	4.5±0.2	1.0×10^−9^
	ATF	3.4±0.4	1.0×10^−9^
IBMX	CREB	4.6±0.1	1.0×10^−9^
	ATF	4.1±0.0	1.0×10^−9^
EHNA	CREB	4.1±0.3	2.2×10^−5^
	ATF	3.6±0.2	2.5×10^−3^
Rolipram	CREB	4.4±0.3	1.0×10^−9^
	ATF	3.6±0.2	1.0×10^−9^
8-bromo-cGMP	CREB	0.3±0.2	5.1×10^−1^
	ATF	0.2±0.2	4.0×10^−1^
Vardenafil citrate	CREB	0.3±0.2	2.5×10^−1^
	ATF	0.7±0.2	5.2×10^−1^
Sildenafil citrate	CREB	0.5±0.2	4.1×10^−1^
	ATF	−0.3±0.2	5.5×10^−1^

HEK293 cells transfected with pool of plasmids (listed in Table S2 and pRL-SV40) and were subsequently treated with chemicals of interest. Values are presented as log2 treatments of the fold induction of the TFBS-directed UR expression after treatment with the inducer of interest. The errors are calculated as 1 standard error of the mean each way. P-values indicate the posterior probability that there was no difference in expression levels between the control and treatment samples so a lower p-value would indicate a greater likelihood that there was a difference between the control and treatment samples. Abbreviations: IBMX: 3-isobutyl-1-methylxanthine, EHNA: erythro-9-(2-hydroxy-3-nonyl)adenine.

**Figure 3:**
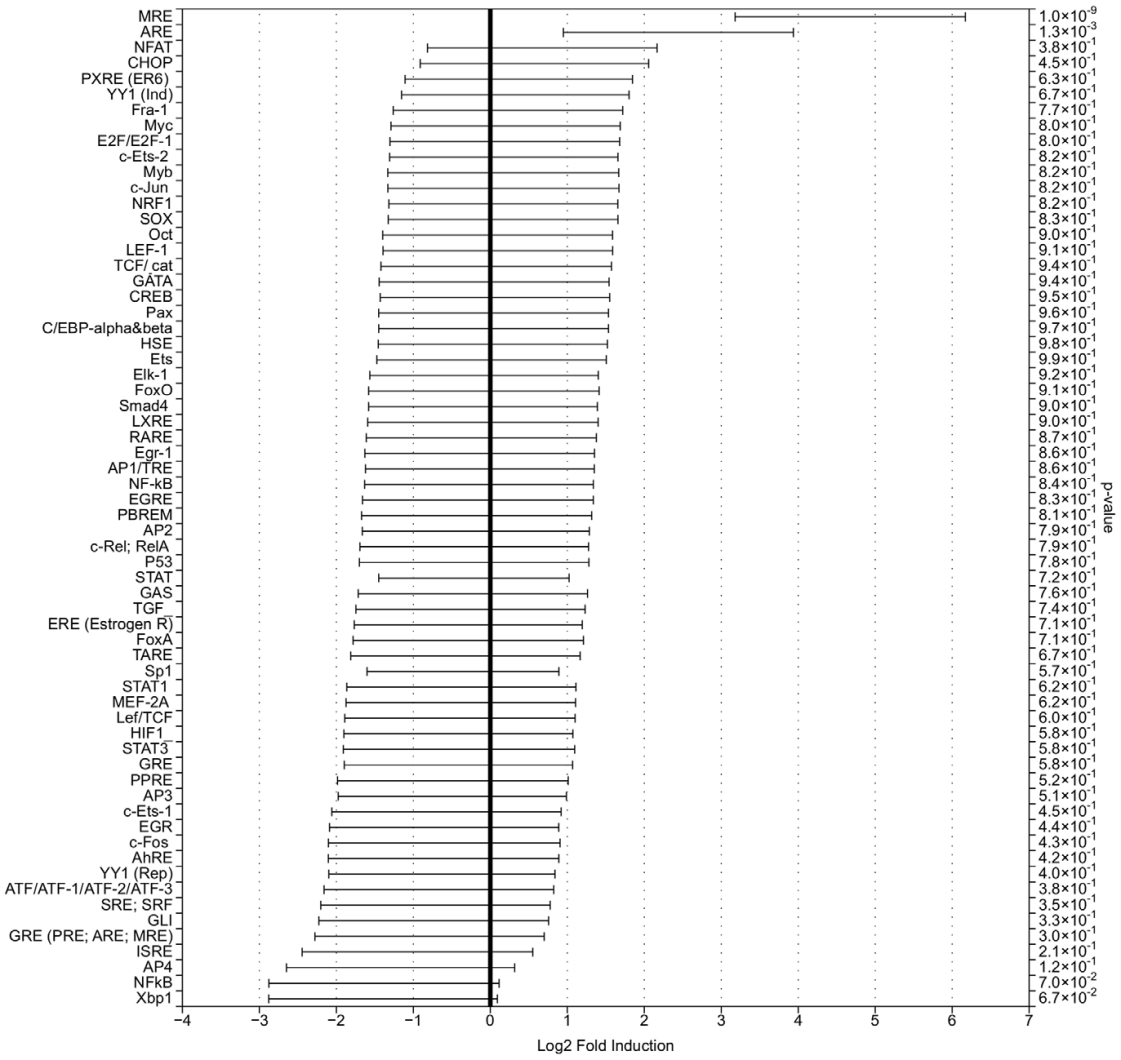
qPCR analysis of induction of TFBS-directed UR expression in treated cells transfected with TFBS-UR plasmids. The statistical model calculated a posterior probability distribution over the mean of the log normalized fold induction. The p-value indicated the posterior probability that there was no difference in expression levels between the control and treatment samples. 95% credible intervals were also calculated for the mean log normalized fold induction and indicate the region where there is a 95% probability that the mean effect lies within it. Bars not crossing the 0 line show significant evidence for an effect following treatment with the inducer of interest.

### Transfection and Treatment of HEK293 Cells

4×10^6^ HEK293 cells were transfected using Genejuice (Novagen), as recommended by the manufacturer, with 800 ng DNA consisting of 11.5 ng pRL-SV40 (Promega) and 11.5 ng of each TFBS-encoding plasmid. After 16 hours, cells were treated with 50 µM CdCl_2,_ 1 µM dexamethasone, 25 µM forskolin or 0.05 µM phorbol-12-myristate 13-acetate (TPA) for 4 hours before mRNA and protein analysis. In the phosphodiesterase inhibitor (PDEI) experiment, transfected HEK293 cells were treated with 25 µM forskolin, 1 mM 8-bromo-cAMP, 1 mM 8-bromo-cGMP, 1 mM 3-isobutyl-1-methylxanthine (IBMX), 1 mM erythro-9-(2-hydroxy-3-nonyl)adenine (EHNA), 1 mM rolipram, 1 mM vardenafil citrate (Sequoia Research Products) or 1 mM sildenafil citrate (Sequoia Research Products) for 2 hours.

Cyclic AMP XP™ and Cyclic GMP XP™ Assay Kits (Cell Signalling) were used to determine the intracellular levels of cAMP and cGMP in HEK293 cells treated with forskolin, cyclic nucleotide analogues or PDEI at the concentrations used above and these were compared to the levels of cAMP and cGMP in untreated cells.

### Western Blots

Protein extracts were separated on Nu-PAGE 4–12% Tris-Acetate acrylamide gels (Invitrogen) in 3-(*N*-morpholino)propane sulfonic acid (MOPS)-sodium dodecyl sulphate (SDS) buffer (Invitrogen). SDS-PAGE and western blotting were performed using standard protocols. Gels were transferred onto Immobilon-P membrane (Millipore). Cell Signalling primary antibodies were used unless otherwise stated: phospho-CREB/phospho-ATF (9198S), CREB/ATF (9197S), SP1 (Santa Cruz antibodies; sc14027), phospho-c-jun (9261S), c-jun (9165S), phospho-IκB (2859S), IκB (4812S) and α-tubulin (Santa Cruz antibodies; sc-8035). Anti-rabbit (Thermo Scientific, 31460) and anti-mouse (Sigma, A4416) peroxidase-conjugated goat secondary antibodies were used. Signal was detected with SuperSignal West Pico Chemiluminescent substrate (Thermo Scientific) using the GBOX/CHEMI-HR16-E-BOX Gel Documentation System (Syngene) and analyzed with the GeneSnap software (Syngene) or ImageJ (http://rsb.info.nih.gov/ij). Membranes were stripped of antibodies and then re-probed for the non-phosphorylated protein or for α-tubulin to verify equal protein loading.

**Figure 4:**
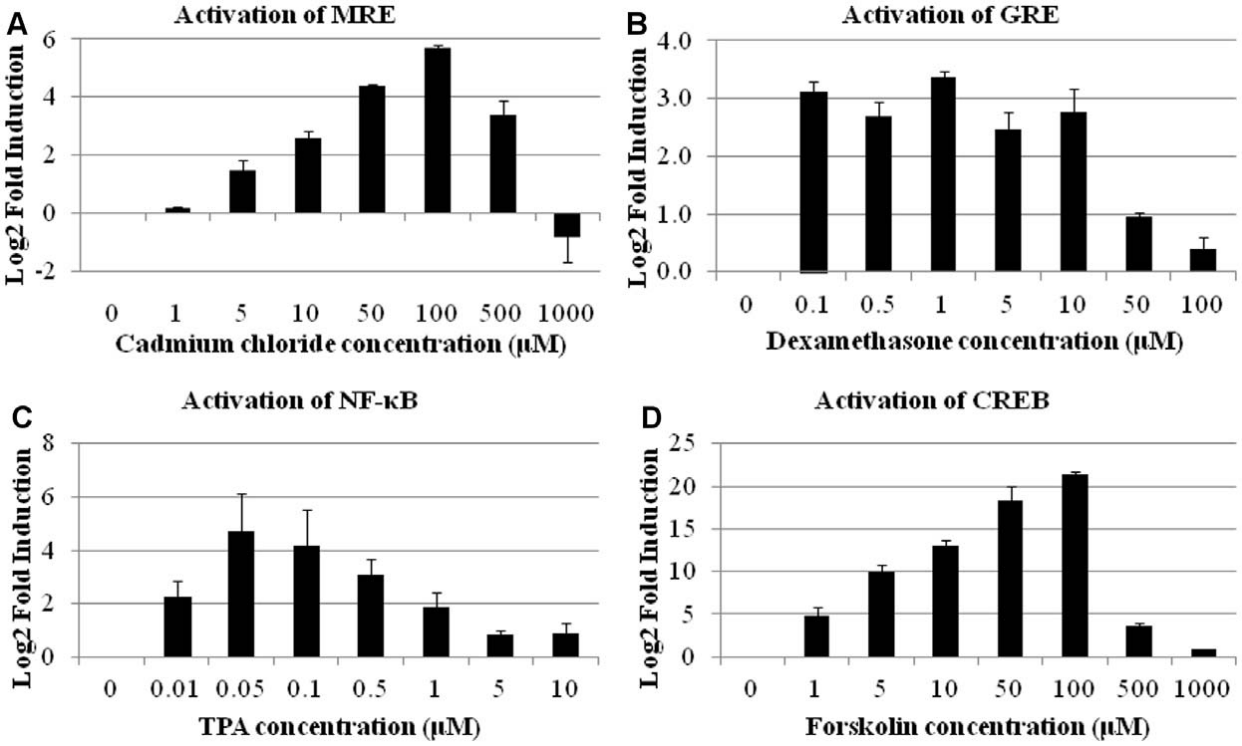
Induction of selected TFBS-directed UR expression in HEK293 cells after treatment with cadmium, dexamethasone, TPA and forskolin. HEK293 cells transfected with a plasmid pool, that included the plasmids listed in Table S2 and pRL-SV40 and were subsequently treated with drugs of interest. (A) MRE-directed UR expression after treatment with cadmium. (B) GRE-directed UR expression after treatment with dexamethasone. (C) NF-κB-directed UR expression after treatment with TPA. (D) CREB-directed UR expression after treatment with forskolin. Values are presented as log2 treatments of the fold induction of the TFBS-directed UR expression after treatment with the inducer of interest. The error bars are calculated as 1 standard error of the mean each way.

**Figure 5:**
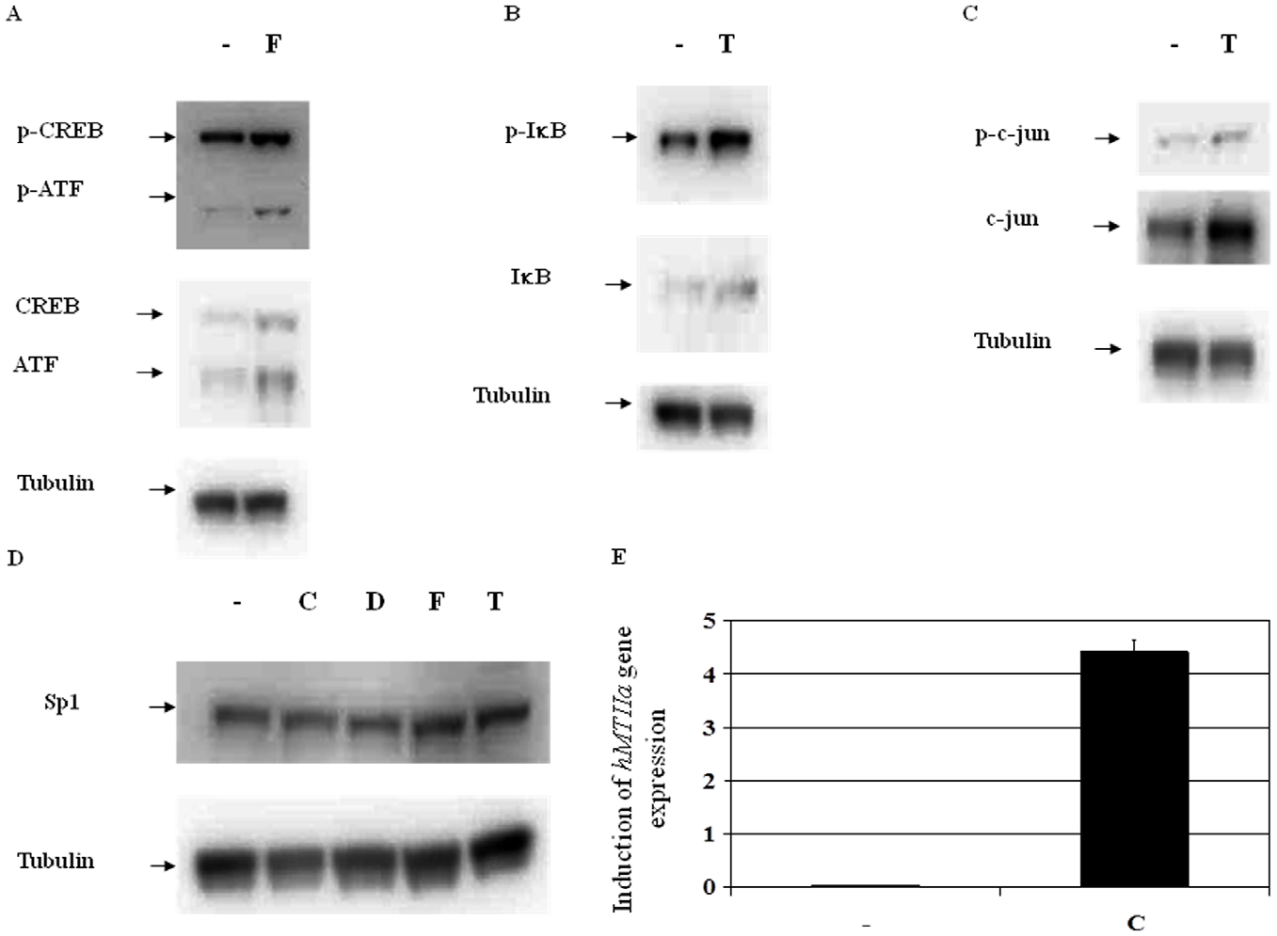
Induction of the TF proteins of interest in HEK293 cells after treatment with forskolin, TPA and cadmium. Proteins extracted from treated and control cells were analyzed using Western blots and TF-specific antibodies. The levels of phosphorylated TFs and inactive TFs were analyzed for (A) CREB and ATF, (B) IκB, (C) c-jun and (D) SP1. Tubulin was used as a loading control. Quantification of the levels of protein on the Western blots showed a 1.6 and 1.3 fold increase in P-CREB and P-ATF after treatment with forskolin and a 1.5 and 1.6 fold increase in P-IκB, and P-c-jun after treatment with TPA. Treatment of HEK293 cells with cadmium chloride, dexamethasone, forskolin and TPA resulted in a 1.1, 1.1. 1.0 and 1.0 fold increase in the levels of SP1 protein. (E) Increased *hMTIIA* gene expression in HEK293 cells after treatment with cadmium. Expression of the cadmium-responsive *hMTIIa* gene was normalized to the expression of the chromosomal reference gene *B2M.* Abbreviations: -, carrier only control; C, cadmium; D, dexamethasone; F, forskolin; T, TPA.

### RNA Purification and cDNA Synthesis

Total RNA was prepared using the miRNeasy mini kit (Qiagen), mRNA was isolated using Dynabeads mRNA Purification Kit (Invitrogen) and was reverse transcribed using Superscript II enzyme (Invitrogen). UR transcripts, expressed from the TFBS-UR encoding plasmids, and Rluc, expressed from pRL-SV40, were analyzed using microarrays and qPCR respectively.

### Microarrays

Unmodified HPLC-purified oligonucleotide captures (Table S2) were diluted to 25 µM in 0.15 M NaH_2_PO_4_ (pH 8.5) and 280 ρL used per spot. 8 subarrays were generated on each epoxy-coated glass slide using the Scienion SciFlexarrayer S3; each subarray included 5 replicates of each UR and Rluc. The fabricated slides were incubated overnight at ambient temperature in 70% relative humidity, pre-hybridised and washed using standard conditions.

Duplicate UR and Rluc PCR reactions were performed for each cDNA sample (3 µL) using Cy3/Cy5-labeled sense and unlabelled antisense primers (2 µM UR primers and 1 µM Rluc primers, Table S1) for 25 cycles in a Mastercycler epgradient PCR machine (Eppendorf) using HotStarTaq DNA polymerase (Qiagen). The fluorescently-labelled amplicons were purified using the QIAquick PCR purification kit (Qiagen), diluted 4 fold with hybridization buffer, denatured for 5 minutes at 95°C, and 4 µL of the diluted amplicons were placed on two successive subarrays which were incubated overnight in a humidified chamber at 53°C. Microarray slides were washed once in 2XSSC, 0.1% SDS at 53°C for 5 minutes, twice in 1XSSC for 2 minutes at room temperature and finally twice in 0.1XSSC for 1 minute at room temperature. The microarray slides were dried by centrifugation at 1,600*×g* for 5 minutes.

Fluorescent images were captured and analyzed with a Perkin-Elmer ScanArray Express scanner and software. Transfection efficiency was accounted for by normalizing the mean signal-background value for each UR to the corresponding signal-background value for Rluc. Changes in gene expression were quantified by calculating the log2 ratio of normalized values for treated cells compared to untreated cells.

### qPCR

UR and Rluc analysis was conducted with Lightcycler Probes Master mix (Roche) in a Lightcycler 480 (Roche). Primer sequences are shown in Tables S1 and S2. A 10 fold dilution series of UR or Rluc linear dsDNA was created and a standard curve generated as described before [20]–[21]. Efficiency of the qPCR reaction (E) was calculated and primer pairs with E = 1.6–2.4 were typically used [22]. Unknown samples were compared to the standard curve and the copy number calculated. Transfection efficiency was accounted for by normalizing the UR copy numbers to that of Rluc in each sample [20]. Changes in gene expression were quantified by comparing the log2 ratio for treated cells to untreated cells.

### Data Normalization

A pool of TFBS-UR encoding reporter plasmids and the control plasmid pRL-SV40 was used to transfect HEK293 cells. Yin *et al*. [23] reported an inverse correlation between plasmid size and the transfection efficiency of that plasmid. As all the reporter plasmids and pRL-SV40 were similar in size, we determined that the transfection efficiencies would not vary significantly between these plasmids. If there were differences in the rate of plasmid uptake between plasmids, these rates would be comparable for the same plasmid between the different biological samples.

We have previously demonstrated that Rluc, expressed from the co-transfected plasmid pRL-SV40, was better as a reference for normalization than the products of many commonly used endogenous chromosomal reference genes [20]. This is because it is stably expressed at levels similar to those of the reporter plasmids and it takes into account all the factors that affect expression within the experiment, including transfection efficiency. The inclusion of a co-transfected control significantly improved the reproducibility and the validity of biological experiments. The use of this Rluc normalization protocol should ensure that any statistically significant differences in TFBS-directed UR expression after sample treatment were a result of the treatment of interest.

### Statistical Analyses

All data was transformed by taking logarithms to the base 2. For microarrays this was the value for the normalized relative fluorescence, and for qPCR, this was the normalized copy number obtained from the Ct values. The log transformed data were analyzed in JAGS [24], a Gibbs sampler for hierarchical models and Coda [25], a tool for examining Markov Chain Monte Carlo runs. For the microarray data, a hierarchical repeated-measures two-way Bayesian ANOVA model was used. For the qPCR data, a three-way Bayesian ANOVA model was used.

## Results and Discussion

Microarray-based analysis required the presence of a unique sequence that could be specifically identified for each signal of interest. In contrast, qPCR requires the presence of three sequences on the reporter; a forward primer, a fluorescently-labelled probe binding site and a reverse primer. To meet the criteria for both these techniques, a DNA reporter was designed with a 23 nucleotide variable unique reporter (UR) region attached to a constant 58 nucleotide sequence (Figure 1). For the microarrays, the variable region was specifically targeted with an anti-sense capture (Table S2). For qPCR, the specific forward primer (Table S2) overlapped the variable region and the fluorescent probe and the reverse primer were located in the constant region, allowing specific qPCR reactions to be conducted for each UR. This design minimized the differences between the URs so the same reaction parameters could be used for all the UR constructs. A program was written to generate UR sequences that were designed to be unique in the human genome, specific, comparable in physicochemical parameters (length, GC content, melting temperatures, with minimal secondary structure and without tetranucleotide runs) and detectable by both qPCR and microarrays (Daly *et al*., manuscript in preparation). Experimental analysis of these automatically generated sequences showed that captures and forward primers could differentiate between URs with a high degree of sequence identity (Arrays: 74%; qPCR: 83%).

HEK293 cells were transfected with plasmids containing the transcription factor binding sites and unique reporters (TFBS-UR), the cells treated under a variety of conditions, mRNA purified and cDNA synthesized. This cDNA was analyzed using both microarray and qPCR-based detection platforms (Figure 1). We routinely observed log2 values between −1 to +1, corresponding to 2 fold repression or induction, for basal levels of TF activity on both detection platforms and attributed these to treatment-independent biological variation in the system. For stringency, data with values between these cut-off points and data with error bars that brought them into this region were not considered to be significant.

Initially, HEK293 cells were treated with cadmium. Both microarray and qPCR platforms showed that cadmium treatment resulted in an increase in activation of metal responsive element (MRE) and antioxidant response element (ARE) directed UR expression (Figures 2A and 2B, Table S3). Cadmium is known to activate the metal responsive (MR) pathway and the MRE [26] and ARE have been reported to bind TFBSs in response to cell stress, especially after exposure to xenobiotics like cadmium [27]–[28]. This information supports the data collected from both detection platforms. No TF activity was significantly down-regulated after cadmium treatment (Figures 2A and 2B, Table S3). Intra-experimental data showed up to 30% variation between biological replicates, and in some cases inter-experimental fold-change values varied up to 60%; however, in both cases the direction of change and the activity profiles remained essentially the same where significant fold change occurred.

A statistical model that calculated a posterior probability distribution over the mean of the log normalized fold induction was used to test the validity of the data. The tails of this posterior distribution were compared to 0 (indicating no induction), to obtain a p-value. This p-value indicated the posterior probability that there was no difference in expression levels between the control and treatment samples. 95% credible intervals were calculated for the mean log normalized fold induction, indicating the region where there is a 95% probability that the mean effect lies within it. Bars not crossing the 0 line show significant evidence for an effect following treatment with the corresponding inducer. The analysis of data generated for cadmium-treated HEK293 cells on the qPCR-based platform (Figure 3) is in agreement with the conclusions drawn from the biological data; cadmium treatment of HEK293 cells has a significant effect on two TFs from the library, MRE and ARE.

Alternative inducers were then used to show the selectivity of the method (Table 1). Treatment with dexamethasone resulted in the activation of two different GREs and the SRE/SRF, consistent with activation of the glucocorticoid response (GR) pathway [29]. Treatment with TPA resulted in increased NF-κB and AP-1 activities; these both have important roles in the cellular proliferation and immune responses respectively and have been shown previously to be induced by TPA [30]–[32]. Forskolin treatment caused an activation of CREB and ATF, two cAMP responsive proteins active in the mitogen-activated protein kinase and G-protein coupled receptor pathways respectively, consistent with an increase in cellular cAMP levels [33]–[34].

To demonstrate that TF activity was related concentrations of inducer, reporters that showed changes in their activity after treatment (MRE, CREB, NF-κB and GRE) were studied at a range of inducer concentrations in HEK293 cells (Figure 4). Cadmium, TPA and forskolin produced generally similar patterns of TF activation with approximately linear response to increasing inducer concentration up to concentrations that became toxic to the cells. MRE activity increased to 100 µM cadmium but decreased above 100 µM (Figure 4A). NF-κB activity increased significantly upon the addition of even low levels of TPA, up to 0.05 µM, but then decreased only gradually to 5 µM, suggesting that either the cell population showed a range of tolerance to TPA toxicity, or that cells were able to adapt to high TPA levels (Figure 4C). Finally, treatment of cells with forskolin resulted in increased CREB activity at concentrations up to 500 µM, above which CREB activity dropped significantly showing marked toxicity (Figure 4D). In contrast, GRE expression induced by dexamethasone showed a rather different profile; GRE activity was induced comparably by the addition of between 0.1 to 10 µM dexamethasone, with no apparent concentration dependence. Concentrations above 10 µM resulted in reduction in the levels of activity due to toxicity (Figure 4B). At concentrations above which the inducers were toxic to the cells, the TF activity profiles changed from inducer-dependent changes in TF activity to toxicity-related changes. When inducers were at concentrations that were toxic to the cell, we observed an increase in the activity of TFs that were indicators of cellular stress responses and apoptosis including p53, STAT3 and c-Jun (data not shown). There was also a dramatic increase in the variation between the biological replicates within the experiment (up to 300%). For studies focused on the effect of inducers on cell signalling, we observed that it was very important to determine the concentrations at which the inducer was toxic to the cell and to conduct studies below this threshold concentration.

To show the specificity of the measured forskolin effect, and the ability of the platform to detect the effects of chemical intervention, the cells were treated with phosphodiesterase inhibitors (PDEIs), which target the enzymes involved in the regulation of cyclic nucleotide metabolism, and are classified as non-specific (N), cAMP-specific (A) or cGMP-specific (G) [35] depending on their action (Figure S1). PDEIs used were selected based on their cyclic nucleotide selectivity and specificity [35], and included IBMX (N) [36]–[37], EHNA (N) [36], rolipram (A) [36]–[38], vardenafil (G) and sildenafil (G) [37], [39]. In addition, cell membrane-permeable stable analogues of cAMP and cGMP were included as internal controls. HEK293 cells were treated with forskolin, 8-bromo-cAMP, 8-bromo-cGMP or PDEI, and the levels of cAMP and cGMP in treated cells were measured and compared to untreated cells. HEK293 cells treated with forskolin, 8-bromo-cAMP, IBMX, EHNA and rolipram showed elevated levels of cellular cAMP and those treated with 8-bromo-cGMP, IBMX, EHNA, vardenafil and sildenafil showed elevated levels of cellular cGMP (Table S4).

The effect of these treatments on TF activity in treated cells was measured on the microarray and qPCR platforms. The data obtained on the two detection platforms was comparable. Treatment of HEK293 cells with 8-bromo-cAMP, non-selective (IBMX, EHNA) or cAMP-specific PDEI (rolipram) resulted in increased CREB and ATF activity (Table 1). In contrast, increased cGMP levels following treatment with 8-bromo-cGMP or cGMP-specific PDEIs (vardenafil and sildenafil), did not result in elevated CREB or ATF activity (Table 1). The differences in the transcriptional readout after PDEI treatment of HEK293 cells allowed us to differentiate between non-specific/cAMP-specific and cGMP-specific PDEI treatments at the TF activity level, opening up significant opportunities for screening new compounds.

To independently validate the changes in TF activity observed on the microarray and qPCR platforms, total protein extracted from treated HEK293 cells was analyzed by western blots. Forskolin-treated HEK293 cells showed increased levels of phospho-CREB and phospho-ATF (Figure 5A). TPA treatment resulted in elevated levels of phospho-IκB, which is known to correlate to phospho-NF-κB in the cell, and a slight increase in phospho-c-jun, which is one of the proteins that constitutes and activates the AP1 TF complex (Figure 5B and 5C). SP1, a TF involved in differentiation, was included as a negative control in this experiment as its levels should be unaffected by the conditions being tested in this report ([40], Figure 5D). Unfortunately, no antibodies were available to study the effect of cadmium on the MR pathway. However, Karin *et al*. [41] have reported that the human metallothionein IIa gene (*hMTIIa*) was responsive to cadmium treatment so *hMTIIa* expression in treated cells was used to confirm activation of the MR. Expression of *hMTIIa* was normalized to *B2M*, the stably-expressed chromosomal gene for beta-2-microglobulin [20]. *hMTIIa* expression increased dramatically (from 0.03±0.01 to 4.41±0.24, Figure 5E) after treatment of HEK293 cells with cadmium, representing a 147 fold increase. These data independently corroborate the biological data collected on the microarray and qPCR detections platforms, confirming that the up-regulation of TFs activity observed on the detection platforms also existed at the protein level.

### Conclusions

Our experimental system exhibits a number of advantages for the analysis of TF activity. The design of the UR ensures that we have access to a large number of reporters, and we are in the process of completing a 1000 component library. The lack of enzymatic labeling steps reduces inherent errors, and the direct analysis of multiplexed PCR reactions and the inclusion of internal normalization controls (e.g. Rluc and pMN222-pMN224) allow the generation of comparative data between biological samples and between independent experiments. The availability of two detection platforms ensures that the data can be validated by two independent systems from the same experiment.

This sensor platform, as with other reporter-based platforms, does suffer from a few technical limitations. Transfection of cells with reporter plasmids is required, and for larger libraries this needs to be performed with a relatively high plasmid concentration; this could in itself influence cellular processes. In addition, transfection of plasmids encoding multiple copies of TFBS could lead to TF sequestration for low copy number TFs, which in turn could affect cellular signalling, although the individual plasmid concentrations are relatively low. Botvinnik *et al*. [13] reported that the use of TFBS-encoding constructs in pools reduced these risks and our data corroborates this observation. However, it would be advisable to validate the results from the multiplexed platform using an alternative system. It is also important to note that, as with any transfection based strategy that for cell lines that are difficult to transfect, transfection efficiency could be low and reduced levels of mRNA transcripts produced, making these reporter systems less suitable for use in these cell lines. With the system reported in this paper it would be possible to concatenate the TFBS-UR reporters and deliver these constructs using viral vectors to overcome these problems, although this requires the development of sufficiently strong insulator elements that could be inserted between the reporters to prevent the results being influenced by read-through from the stronger TFs. One last technical limitation is linked to the high throughput microarray-based detection platform. This analysis currently requires PCR amplification and labelling of the UR transcripts before detection. This necessitates some optimization of PCR conditions, and it is difficult to analyze TFs across a broad range of activities in the same experiment, as it is likely that weak signals may not be detected. To address this, the array analysis was performed on samples from three sets of PCR reactions generated at increasing cycle numbers to ensure that signal was detected from low level transcripts while still maintaining accurate analysis of high level transcripts.

Our proof of concept biological treatments raises interesting applications for this technology from studying signalling pathways of interest to building and testing inferential models of signalling to screening drugs for desired therapeutic or undesired off-target effects. The system is straightforward, robust, and allows more information to be extracted from each experiment, a factor that is increasingly important considering the time and cost of biological studies.

## Supporting Information

Figure S1 **Schematic representation of the cAMP and cGMP signalling pathways in mammalian cells.** The cyclic nucleotides cAMP and cGMP are generated by the activation of membrane-bound receptors coupled to AC or GC. cAMP stimulates the cAMP-sensitive PDE and PKA both of which result in the stimulation of cAMP-dependent cellular responses including the activation of the TFs CREB and ATF. cGMP activates PKG and the cGMP-dependent PDE which in turn activates the cGMP dependent cellular pathways. IBMX inhibits non-specific PDE 1, EHNA inhibits cAMP- and cGMP-specific PDE2, rolipram inhibits cAMP-specific PDE4 and both vardenafil and sildenafil inhibit cGMP-specific PDE5. Abbreviations: AC: adenylyl cyclase, GC_P_: guanylyl cyclase (particulate), GC_S_: guanylyl cyclase (soluble), EHNA: erythro-9-(2-hydroxy-3-nonyl)adenine, IBMX: 3-isobutyl-1-methylxanthine, PKA: protein kinase A, PKG: protein kinase G.
(TIF)

Table S1 **Primers used in this study.**
(DOCX)

Table S2 **List of plasmids in the unique reporter-based sensor platform library to date.**
(DOCX)

Table S3 **Data for ‘Analysis of induction in cadmium chloride-treated cells transfected with TFBS-UR plasmids’.** HEK293 cells transfected with a plasmid pool, that included the plasmids listed in Table S2 and pRL-SV40 and were subsequently treated with cadmium. (A) Microarray-based detection of TF derived activation of UR expression. (B) qPCR-based detection of TF-derived activation of UR expression. Values are presented as log2 treatments of the fold induction of the TFBS-directed UR expression after treatment with the inducer of interest. TFBS marked with in red represent treatment-dependent effects on the TF library. The SEM values are calculated as 1 standard error of the mean each way.
(DOCX)

Table S4 **Changes in the levels of cAMP and cGMP in HEK293 cells treated with cyclic nucleotide analogues or PDEI.** HEK293 cells transfected with pool of plasmids (listed in Table S2 and pRL-SV40) and were subsequently treated with chemicals of interest. Levels of intracellular cAMP and cGMP were quantified and are presented as the increase in the intracellular cAMP or cGMP levels in cells treated with inducers of interest compared to the levels in untreated cells. The errors are calculated as 1 standard error of the mean each way. Abbreviations: IBMX: 3-isobutyl-1-methylxanthine, EHNA: erythro-9-(2-hydroxy-3-nonyl)adenine.
(DOCX)
