# Supplementary figures and images for: Unique Reporter-Based Sensor Platforms to Monitor Signalling in Cells
Source: PLoS One. 2012 Nov 29;7(11):e50521. doi: 10.1371/journal.pone.0050521 (PMC3510088; doi:10.1371/journal.pone.0050521)

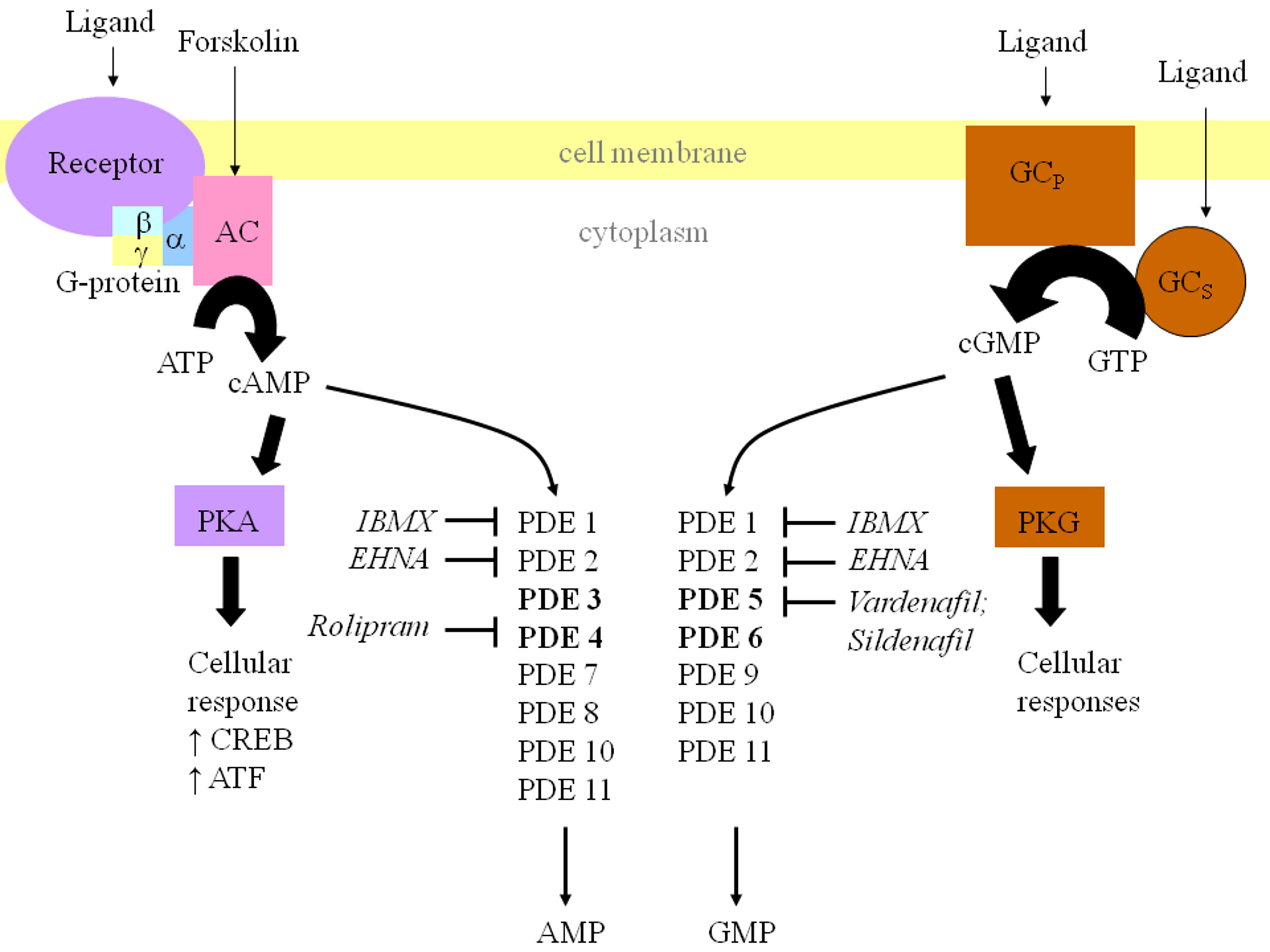

Supplement: Figure S1 — Schematic representation of the cAMP and cGMP signalling pathways in mammalian cells. The cyclic nucleotides cAMP and cGMP are generated by the activation of membrane-bound receptors coupled to AC or GC. cAMP stimulates the cAMP-sensitive PDE and PKA both of which result in the stimulation of cAMP-dependent cellular responses including the activation of the TFs CREB and ATF. cGMP activates PKG and the cGMP-dependent PDE which in turn activates the cGMP dependent cellular pathways. IBMX inhibits non-specific PDE 1, EHNA inhibits cAMP- and cGMP-specific PDE2, rolipram inhibits cAMP-specific PDE4 and both vardenafil and sildenafil inhibit cGMP-specific PDE5. Abbreviations: AC: adenylyl cyclase, GCP: guanylyl cyclase (particulate), GCS: guanylyl cyclase (soluble), EHNA: erythro-9-(2-hydroxy-3-nonyl)adenine, IBMX: 3-isobutyl-1-methylxanthine, PKA: protein kinase A, PKG: protein kinase G. (TIF) [file pone.0050521.s001.tif]
