# Supplementary material for: Unique Reporter-Based Sensor Platforms to Monitor Signalling in Cells
Source: PLoS One. 2012 Nov 29;7(11):e50521. doi: 10.1371/journal.pone.0050521 (PMC3510088; doi:10.1371/journal.pone.0050521)
Supplement: Table S1 — Primers used in this study. (DOCX) [file pone.0050521.s002.docx]

**Table S1: Primers used in this study.**

| **Primer** | **Purpose** | **Description** | **Sequence (5' to 3')** |
| --- | --- | --- | --- |
| prMJ183 | S | pMN2 and its derivatives | GCCTCTTCGCTATTACGCCAG |
| prMJ245 | P/qPCR | UR fluorescent probe (5' FAM, 3' BHQ-1) | ctgagtgagatgagcGTGCGAGAGTGCCA |
| prMJ264 | P/qPCR | UR R, Array PCR UR anti-sense | TCTCCGACCGACATCTG |
| prMJ274 | P/qPCR | Rluc F | gcagaagttggtcgtgagg |
| prMJ272 | P/qPCR | Rluc fluorescent probe (5’ HEX, 3’ BHQ-1) | Ctcactataggctagccaccatgacttcgaaag |
| prMJ276 | P/qPCR | Rluc R | tcatccgtttcctttgttctg |
| prMJ313 | P/qPCR | hMTIIa F | gtgggctgtgccaagtgt |
| prMJ314 | P/qPCR | hMTIIa fluorescent probe (5' FAM, 3' BHQ-1) | cgacttccacaaacctggat |
| prMJ315 | P/qPCR | hMTIIa R | atagcaaacggtcacggtca |
| prMJ348 | P/qPCR | B2M F | TCTCTGCTCCCCACCTCTAA |
| prMJ349 | P/qPCR | B2M fluorescent probe (5’ FAM, 3’ BHQ-1) | CCAGCCCTCCTAGAGCTACC |
| prMJ350 | P/qPCR | B2M R | ATCTGAGCAGGTTGCTCCAC |
| AG_Cap Rluc | C | Rluc anti-sense capture | GTTATCATCCGTTTCCTTTGTTCTGG |
| AG_URF | P | Array PCR UR Sense | Cy3/Cy5-GCACCTCTTCGGCAAG |
| AG_RlucF | P | Array PCR Rluc Sense | Cy3/Cy5-Ctcccagttcaattacagctc |
| AG_RlucR | P | Array PCR Rluc anti-sense | ACCAGTTATCATCCGTTTCC |

Abbreviations- C: Capture for DNA microarray, F: forward primer, P: PCR, qPCR: quantitative real-time PCR, R: reverse primer, S: sequencing primer, UR: unique reporter.
