# Supplementary material for: Unique Reporter-Based Sensor Platforms to Monitor Signalling in Cells
Source: PLoS One. 2012 Nov 29;7(11):e50521. doi: 10.1371/journal.pone.0050521 (PMC3510088; doi:10.1371/journal.pone.0050521)
Supplement: Table S2 — List of plasmids in the unique reporter-based sensor platform library to date. (DOCX) [file pone.0050521.s003.docx]

**Table S2: List of plasmids in the unique reporter-based sensor platform library to date.**

| Plasmid | TF | Role | Sequence | Repeats | Sequence ID | Reference | Unique reporter sequence | Microarray anti-sense capture sequence | Specific F qPCR primer sequence |
| --- | --- | --- | --- | --- | --- | --- | --- | --- | --- |
| pMN133 | AhRE | xenobiotic response, hypoxia | TCTCACGCAA | 3x | R00272 | [12] | AGAGTCGCCCATAGCAAACGCCC | GCGTTTGCTATGGGCGACTCT | CCCATAGCAAACGCCC |
| pMN213 | AP-1/TRE | JNK pathway, stress responses, differentiation, proliferation, apoptosis | ATGAGTCAG | 6x | R03533; T00029 | [12] | CGAGTGGTGTTTGGGTGTCGGAA | TTCCGACACCCAAACACCACTC | CGAGTGGTGTTTGGGTG |
| pMN214 | AP-2 | embryonic morphogenesis | GCCCAGGGCt | 3x | R01055 | [12] | TCATGCGGTAAGGCTGGTTGAAC | TTCAACCAGCCTTACCGCATG | GCGGTAAGGCTGGTTG |
| pMN215 | AP-3 | IL-2, Notch signalling | tgtggaaag | 5x |  | [41] | CCCGACCCTGCACCCGTTTTGAT | ATCAAAACGGGTGCAGGGTCG | GACCCTGCACCCGTTT |
| pMN216 | AP-4 | proliferation | [T/C]CAGCTG[T/C]GG |  |  | [42] | AGACGCTTGGTTTGTGCTTCAAT | TGAAGCACAAACCAAGCGTCTG | CGCTTGGTTTGTGCTT |
| pMN135 | ARE | antioxidative responses | AATCGCAGTCACAGTGACTCAGCAGAATCTGAG | 2x |  | [12] | GTCGAGGCAGGTAGAGGATCTCA | ATCCTCTACCTGCCTCGACGC | CGAGGCAGGTAGAGGA |
| pMN217 | ATF/ATF-1/ATF-2/ATF-3 | cAMP/Ca dependent activator protein, | CCATGACGTCAATT | 3x |  | [43] | TGGAGGGCTTCGTTGAAACTGGT | CCAGTTTCAACGAAGCCCTCCA | TGGAGGGCTTCGTTGA |
| pMN185 | C/EBP-α&β | adipogenesis; CCAAT-enhancer binding protein | ATTGCGCAAT | 3x | R00207; T00105 | [12] | TTCTATCCCACGCTGACCTTTGG | CCAAAGGTCAGCGTGGGATAGAA | TCCCACGCTGACCTTT |
| pMN186 | CHOP | ER stress pathways | AGATGCAATCCCC |  |  | [44] | CTACGCGTTGACTTCCCTTATTG | CAATAAGGGAAGTCAACGCGTAG | CGCGTTGACTTCCCTT |
| pMN218 | CREB | cAMP response element; cAMP and cGMP, NO receptor, GPCR pathways | AGCCTGACGTCAGAG | 4x |  | [45] | CCCGGCTGCTCAACCCACTTTCG | CGAAAGTGGGTTGAGCAGCCG | GCTGCTCAACCCACTTT |
| pMN187 | E2F/E2F-1 | cell cycle, proliferation | TTTGGCGC | 2x | R08808; T01542 | [12] | AGGCTGCTTTCTCCCTGCATCTT | AAGATGCAGGGAGAAAGCAGCC | AGGCTGCTTTCTCCCT |
| pmN188 | EGR | early growth response | GCGGGGGCG |  |  | [46] | GGGCTCCGCAACTCCAAGAACGA | TCGTTCTTGGAGTTGCGGAGC | GCTCCGCAACTCCAAG |
| pMN189 | Egr-1 | early growth response factor 1 | GGGGTGGGGN | 3x |  | [47] | TTAGTGCCTGCCTCAAGTGAGTG | CACTCACTTGAGGCAGGCACTAA | AGTGCCTGCCTCAAGT |
| pMN190 | EGRE | receptor tyrosine kinase pathway | CGCCCCCGC | 2x | R02147 | [12] | CTACAATGGCGAGGTAAGTGGCT | AGCCACTTACCTCGCCATTGTAG | GGCGAGGTAAGTGGCT |
| pMN191 | Elk-1 | cell proliferation and differentiation | CCATGGAGGG |  | T00250 | [48-49] | CCCGCGTGCCCGTGTTCCTTTTT | AAAAAGGAACACGGGCACGC | GTGCCCGTGTTCCTTT |
| pMN137 | ERE | nuclear hormone receptor pathway | AGGTCACAGTGACCT | 2x | R02715 | [12] | CAACTTCCCACACCGAATCGCGT | GCGATTCGGTGTGGGAAGTTG | CAACTTCCCACACCGAA |
| pMN141 | Ets | MAP kinase mediated signalling | GGAGGAAGT | 3x | R04051 | [12] | GCGCTCCCTCTGTTGCGCTCCCT | AGGGAGCGCAACAGAGGGAG | CTCTGTTGCGCTCCCT |
| pMN192 | c-Ets-1 | cell proliferation and differentiation | CTTCCG |  | T00112 | [48-49] | TGGTTGCACTACCCTGCCTCCGT | ACGGAGGCAGGGTAGTGCAAC | ACTACCCTGCCTCCGT |
| pMN139 | c-Ets-2 | cell proliferation and differentiation | CACTTCCTG |  | T00113 | [48-49] | GTTAGCGCCGGACTAGGGTGAGC | GCTCACCCTAGTCCGGCGCT | TTAGCGCCGGACTAGG |
| pMN193 | c-Fos | differentiation, proliferation, apoptosis | GACTCA |  | T00123 | [48-49] | CCCTGCCCCGCTATTCTCCGCAC | CGGAGAATAGCGGGGCAGG | CCGCTATTCTCCGCAC |
| pMN143 | FoxA | hepatic specification | GAGTGTTTACTT | 3x | R02177 | [12] | GGGTCCACACTGAAGCCCGACTT | AGTCGGGCTTCAGTGTGGACC | GTCCACACTGAAGCCC |
| pMN194 | FoxO | Akt/Forkhead signalling | AGCAAAACAA | 3x | R04663 | [12] | TGCCGGTACGTGCGTCATGGGGT | ACCCCATGACGCACGTACCG | GTACGTGCGTCATGGG |
| pMN145 | Fra-1 | IL-6/JAK/Stat3 | TGAGTCAT |  | T01462 | [48-49] | TGCTCGGGCCTGGTCTTTATCCT | GATAAAGACCAGGCCCGAGCA | CTCGGGCCTGGTCTTT |
| pMN195 | GAS | interferon gamma-activated sequence | AGTTTCATATTACTCTAAATC | 4x |  | [47] | GCCGCCCAGAAGCACGAAATGTG | ACATTTCGTGCTTCTGGGCGG | GCCCAGAAGCACGAAA |
| pMN211 | GATA | differentiation | cTGATAa | 3x | R00507 | [12] | ATGCATACATCGCCCTACTCGCT | AGCGAGTAGGGCGATGTATGCA | CATCGCCCTACTCGCT |
| pMN149 | GLI | hedgehog pathway | GACCACCCA | 3x | R01930 | [12] | TTATTTAAGGACTTGGCGCACTC | GAGTGCGCCAAGTCCTTAAATAAA | AAGGACTTGGCGCACT |
| pMN182 | GRE [PRE, ARE, MRE] | differentiation, inflammation | AGAACAGGATGTTCT | 3x | R03199 | [12] | CGTCGTCGCTGGGTGTGTTTGTC | ACAAACACACCCAGCGACGACG | CGCTGGGTGTGTTTGT |
| pMN219 | GRE | differentiation, inflammation | TGTACAGGATGTTCT | 4x |  | [48-49] | TTCCGACGGTGCTCTTCTTGTCG | ACAAGAAGAGCACCGTCGGAA | GACGGTGCTCTTCTTGT |
| pMN151 | HIF1α | hypoxia-inducible factor | GCCCTACGTGCTGTCTCACACAGCCTGTCTGACCTCTCGACCTACCGGCC |  |  | [50] | AATTGTGGCAACTTACGTCCCGA | TCGGGACGTAAGTTGCCACAAT | GGCAACTTACGTCCCG |
| pMN153 | HSE | stress response, heat shock | CtcGAAtgTTCgcG | 2x | R00756 | [12] | AGTCTACCGAGTTGCCCTCTCTG | AGAGGGCAACTCGGTAGACTGGA | GTCTACCGAGTTGCCCT |
| pMN212 | ISRE | immune responses, host defense | CTAGTTTCACTTTCCC | 5x | R00950 | [12] | AACAAATTGGGCGAGGGTGTGCA | TGCACACCCTCGCCCAATTT | ATTGGGCGAGGGTGTG |
| pMN155 | c-Jun | differentiation, proliferation, apoptosis | TGAGTCA |  | T00133 | [48-49] | GGCTTCCATTCCCGTTTCCCTGT | AGGGAAACGGGAATGGAAGCC | GGCTTCCATTCCCGTTT |
| pMN196 | LEF-1 | wnt signalling | CTTTGAT |  | T02905 | [48-49] | GAATTCGTTCTCTCCGCCTTGAC | GTCAAGGCGGAGAGAACGAATT | CGTTCTCTCCGCCTTG |
| pMN223 | Lef/TCF | wnt signalling | GCACCCTTTGAAGCTC | 7x |  | [51] | GGGTATATATGCGCCGGGTTGCA | GCAACCCGGCGCATATATACC | TATATGCGCCGGGTTG |
| pMN157 | LXRE | nuclear hormone receptor pathway | AGGTCATCTCAGGTCA | 2x | R10745 | [12] | TTGGGTTTCACGGGTTATGGACA | TGTCCATAACCCGTGAAACCCAA | TTGGGTTTCACGGGTT |
| pMN197 | MEF-2A | CaMK and MAPK mediated signalling | CTAAAAATAGC |  | T01005 | [48-49] | TGGCGCATCCCAAGCACTAAGAG | CTCTTAGTGCTTGGGATGCGCC | CGCATCCCAAGCACTAA |
| pMN220 | MRE | cell stress response, metallothioneins, metal transport | CGGGTGCGCCCGGCCCGA | 5x |  | [52] | CACGGACTCGTGCTATGCCGTCC | GACGGCATAGCACGAGTCCG | TCGTGCTATGCCGTCC |
| pMN159 | Myb | hematopoietic cell differentiation | CAACCGTTAT | 3x | R01431 | [12] | TAATGATGCGTCCACTTGCTTGT | ACAAGCAAGTGGACGCATCATTA | ATGCGTCCACTTGCTT |
| pMN198 | Myc | cell cycle, proliferation | CACGTG | 6x | R02207 | [12] | ACGCCTCTAAACCGCAATAGTGA | TCACTATTGCGGTTTAGAGGCGT | CGCCTCTAAACCGCAA |
| pMN183 | NFAT | nuclear factor of activated T-cells | GGAGGAAAAACTGTTTCATACAGAAGGCGT | 4x |  | [47] | AGCTTGCGTGCTCTACTTGAAGT | GACTTCAAGTAGAGCACGCAAGCT | GCTTGCGTGCTCTACTT |
| pMN199 | NF-κB | immune responses, IL-1/Toll receptor pathway | GGGAATTTCC | 4x | R03342 | [12] | GACCAAAGCACAAACCCTCTTCG | CGAAGAGGGTTTGTGCTTTGGTC | GACCAAAGCACAAACCCT |
| pMN221 | NF-κB | immune responses, IL-1/Toll receptor pathway | Tggggactttccgc | 5x |  | [53] | GGCACACCGCCAATAGTTCCAGG | CCTGGAACTATTGGCGGTGTGC | ACACCGCCAATAGTTCC |
| pMN200 | NRF1 | mitochondria genesis, antioxidative responses | TGCGCCTGCGCA | 1x | R02670 | [12] | TGAGGGTCTCCGCCTCTCTTTGT | ACAAAGAGAGGCGGAGACCCT | TCTCCGCCTCTCTTTGT |
| pMN161 | Oct | development of CNS, beta cell differentiation | ATGCAAAT | 3x | R00833 | [12] | GCTGGGCTCGCAATAAGGAGCCA | CTCCTTATTGCGAGCCCAGC | TGGGCTCGCAATAAGG |
| pMN184 | P53 | genotoxic stress responses, check point controls | GGACATGCCCGGGCATGTCC | 1x | R04099 | [12] | CGTGACTGCTTCCCTCCTCAAAT | ATTTGAGGAGGGAAGCAGTCACG | TGACTGCTTCCCTCCT |
| pMN163 | Pax | development of CNS, beta cell differentiation | TTCACGCCTGACTGA | 2x | R08704 | [12] | CCCACCTGTTCCTTCCGTGCTTT | AAGCACGGAAGGAACAGGTGGG | CTGTTCCTTCCGTGCTTT |
| pMN165 | PBREM | nuclear hormone receptor pathway | ACTGTACTTTCCTGACCTTG | 2x |  | [12] | TTACTTGCGTGGTGTGAAGCGGA | TCCGCTTCACACCACGCAAGTAA | ACTTGCGTGGTGTGAA |
| pMN167 | PPRE | nuclear hormone receptor pathway | TGACCTTTGTCCT | 3x | R03986 | [12] | CCGCTCCGCCCTTCTCCAAAGAC | TTTGGAGAAGGGCGGAGCG | TCCGCCCTTCTCCAAA |
| pMN169 | PXRE [ER6] | nuclear receptor pathway | CAATATGAACTCAAAGGAGGTCAGTG | 3x |  | [54] | TGATTCCCGCCGTTTGTCGTACG | GTACGACAAACGGCGGGAATCA | TGATTCCCGCCGTTTG |
| pMN201 | RARE | nuclear hormone receptor pathway | AGGTCAtcctcAGGTCA | 2x | R03933 | [12] | GCACCTCCCGAAACACACATTCG | GAATGTGTGTTTCGGGAGGTGC | GCACCTCCCGAAACAC |
| pMN202 | c-Rel; RelA | inflammatory and immune responses | GGGAATTTCC |  | T00168 | [48-49] | GTCCGTCGGTTGACTCGTGCTGG | CAGCACGAGTCAACCGACGGA | GGTTGACTCGTGCTGG |
| pMN203 | Smad4 | cell growth, cell differentiation, apoptosis | AGGCAGAC |  | T04292 | [48-49] | CCGACCCGCTTTCCCACACAACG | GTTGTGTGGGAAAGCGGGTCG | CCGCTTTCCCACACAA |
| pMN171 | SOX | chondrogenesis | CTAGTCCCTGCATAGAACAATGGAGT | 2x | R09327 | [12] | GTCCCGTGCCCTAAACATTCCTA | TAGGAATGTTTAGGGCACGGGAC | TCCCGTGCCCTAAACA |
| pMN227 | Sp1 | differentiation | GAGGCGGAGC | 6x |  | [55] | TGTACCGACGAAGGATTGGATTC | GAATCCAATCCTTCGTCGGTACA | CCGACGAAGGATTGGATT |
| pMN204 | Sp1 | differentiation | GGGCGGGGCG | 3x | R04813 | [12] | CCCAGGCGGCATAACACTTTTAC | AAAAGTGTTATGCCGCCTGGG | CCAGGCGGCATAACAC |
| pMN205 | SRE; SRF | serum response element; factor | AGGATGTCCATATTAGGACATCT | 5x |  | [42] | GGCTAACACCACCCTCTAAAGCT | AGCTTTAGAGGGTGGTGTTAGCC | GGCTAACACCACCCTCT |
| pMN206 | STAT | Jak pathway | TTCTGGGAA | 3x | R04252 | [12] | GACCGGTCCAAGTAGCCCGTATT | AATACGGGCTACTTGGACCGG | GGTCCAAGTAGCCCGT |
| pMN173 | STAT | Jak pathway | TTCTGGGAA | 3x | R04252 | [12] | TAATGGTGTAGCGGCCTTAAAAG | GCTCGCTTGTGGGTAGGAACG | TCCTACCCACAAGCGA |
| pMN175 | STAT1 | Jak pathway | TTCCGGGAA |  | T04759 | [48-49] | GGGCAGTCCGCCACTCCACCTAA | TTAGGTGGAGTGGCGGACTGC | CCGCCACTCCACCTAA |
| pMN207 | STAT3 | cell growth and apoptosis | TTCCCGGAA |  | T01493 | [48-49] | CACGTACAGCGTCCTACTCGTTT | AAACGAGTAGGACGCTGTACGTG | ACAGCGTCCTACTCGTT |
| pMN208 | TARE | TGF-beta/activin response element | CATTGTCAGTCTAGACATACTCCGAGATTGTGGATTGAGA | 3x |  | [47] | GCATTGGGTTCGTGGCGTAGGAT | ATCCTACGCCACGAACCCAATG | ATTGGGTTCGTGGCGT |
| pMN209 | TCF/β-cat | cell adhesion, wingless-Int pathway | CCTTTGATCTT | 6x |  | [12] | CTGCTCGCACGAGAACCACCTTC | AAGGTGGTTCTCGTGCGAGCAG | GCACGAGAACCACCTT |
| pMN210 | TGFβ | cell growth and differentiation TGFb pathway | AGCCAGACA |  |  | [12] | TGTAAGCAACTCCCGATACCCGA | TCGGGTATCGGGAGTTGCTTA | AGCAACTCCCGATACCC |
| pMN177 | Xbp1 | unfolded protein response, ER stress | TGACGTGG | 4x |  | [12] | TGATCCCTCAACCGCACTTGGAT | CCAAGTGCGGTTGAGGGATC | ATCCCTCAACCGCACT |
| pMN179 | YY1 [Ind] | embryogenesis, differentiation, replication and proliferation | gcccggccatcttgtctgct |  |  | [56] | GCGCTTGTCCCGCTTCCTTCATA | TATGAAGGAAGCGGGACAAGCG | GCTTGTCCCGCTTCCT |
| pMN181 | YY1 [Rep] | embryogenesis, differentiation, replication and proliferation | gatgtccatattaggac |  |  | [56] | GAACCCGAACGCAACCAACTTAG | CTAAGTTGGTTGCGTTCGGGTTC | GAACCCGAACGCAACC |
| pMN224 | No TFBS |  |  |  |  |  | CTGACGCGTCCTGTCTTCGTTCA | GAACGAAGACAGGACGCGTCAG | GCGTCCTGTCTTCGTT |
| pMN225 | No TFBS |  |  |  |  |  | TCGCGTCATCCTCCACAATTCAG | CTGAATTGTGGAGGATGACGCG | GCGTCATCCTCCACAA |
| pMN226 | No TFBS |  |  |  |  |  | TGACCCACTAGCCACGAATGGAG | CTCCATTCGTGGCTAGTGGGTCA | ACCCACTAGCCACGAA |

References: (12) Romanov S, Medvedev A, Gambarian M, Poltoratskaya N, Moeser M, Medvedeva L, Gambarian M, Diatchenko L, Makarov S (2008) Homogenous reporter system enables quantitative functional assessment of multiple transcription factors. Nature Methods 5: 253-260. (41) Mercurio F, Karin M (1989) Transcription factors AP-3 and AP-2 interact with the SV40 enhancer in a mutually exclusive manner. EMBO J 8: 1455-1460 (42) Ghosh D (1993) Status of the transcription factors database (TFD). Nucleic Acids Res 21: 3117-3118. (43) Lin Y-S, Green MR (1988) Interaction of a common cellular transcription factor, ATF, with regulatory elements in both Ela- and cyclic AMP-inducible promoters. Proc Natl Acad Sci USA 85: 3396-3400. (44) Ubeda M, Wang X-Z, Zinszner H, Wu I, Habener JF, Ron D (1996) Stress-Induced Binding of the Transcription Factor CHOP to a Novel DNA Control Element. Mol Cellular Biol 16: 1479-1489. (45) Roesler WJ, Vandenbark GR, Hanson RW (1988) Cyclic AMP and the Induction of Eukaryotic Gene Transcription. J Biol Chem 263: 9063-906. (46) Skerka C, Decker EL, Zipfel PF (1997) Coordinate expression and distinct DNA-binding characteristics of the four EGF-zinc finger proteins in Jurkat T lymphocytes. Immunobiology 198: 179-191. (47) Stratagene (2006) PathDetect^®^ in vivo signal transduction pathway cis-reporting systems, Revision A, USA. (48) Messeguer X, Escudero R, Farre D, Nunez O, Martinez J, Alba MM (2002) PROMO: detection of known transcription regulatory elements using species-tailored searches. Bioinformatics 18: 333-334. (49) Farre D, Roset R, Huerta M, Adsuara JE, Rosello L, Alba MM, Messeguer X (2003) Identification of patterns in biological sequences at the ALGGEN server: PROMO and MALGEN. Nucleic Acids Res 31: 3651-3653. (50) Semenza GL, Wang GL (1992) A Nuclear Factor Induced by Hypoxia via De Novo Protein Synthesis Binds to the Human Erythropoietin Gene Enhancer at a Site Required for Transcriptional Activation. Mol Cellular Biol 12: 5447-5454. (51) Hsu S-C, Galceran J, Grosschedl R (1998) Modulation of Transcriptional Regulation by LEF-1 in Response to Wnt-1 Signalling and Association with β-Catenin. Mol Cellular Biol 18: 4807-4818. (52) Stuart GW, Searle PF, Palmiter RD (1985) Identification of multiple metal regulatory elements in mouse metallothionein-I promoter by assaying synthetic sequences. Nature 317: 828-831. (53) Lenardo MJ, Fan C-M, Maniatis T, Baltimore D (1989) Gene Regulation of NF-κB in β-interferon gene regulation Reveals Its Role as Widely Inducible Mediator of Signal Transduction. Cell 57: 287-294. (54) Lehmann JM, McKee DD, Watson MA, Willson TM, Moore JT, Kliewer SA (1998) The Human Orphan Nuclear Receptor PXR Is Activated by Compounds That Regulate CYP3A4 Gene Expression and Cause Drug Interactions. J Clin Invest 102: 1016-1023. (55) Ishii S, Kadonaga JT, Tjian R, Brady JN, Merlino GT, Pastan I (1986) Binding of the Spl transcription factor by the human Harvey rasl proto-oncogene promoter. Science 232: 1410-1413. (56) Shrivastava A, Calame K (1994) An analysis of genes regulated by the multi-functional transcriptional regulator Yin Yang-1. Nucleic Acids Res 22: 5151-5155.
