# Supplementary material for: Unique Reporter-Based Sensor Platforms to Monitor Signalling in Cells
Source: PLoS One. 2012 Nov 29;7(11):e50521. doi: 10.1371/journal.pone.0050521 (PMC3510088; doi:10.1371/journal.pone.0050521)
Supplement: Table S3 — Data for ‘Analysis of induction in cadmium chloride-treated cells transfected with TFBS-UR plasmids’. HEK293 cells transfected with a plasmid pool, that included the plasmids listed in Table S2 and pRL-SV40 and were subsequently treated with cadmium. (A) Microarray-based detection of TF derived activation of UR expression. (B) qPCR-based detection of TF-derived activation of UR expression. Values are presented as log2 treatments of the fold induction of the TFBS-directed UR expression after treatment with the inducer of interest. TFBS marked with in red represent treatment-dependent effects on the TF library. The SEM values are calculated as 1 standard error of the mean each way. (DOCX) [file pone.0050521.s004.docx]

**Table S3: Data for ‘Analysis of induction in cadmium chloride-treated cells transfected with TFBS-UR plasmids’.** HEK293 cells transfected with a plasmid pool, that included the plasmids listed in Supporting Table 2 and pRL-SV40 and were subsequently treated with cadmium. (A) Microarray-based detection of TF derived activation of UR expression. (B) qPCR-based detection of TF-derived activation of UR expression. Values are presented as log2 treatments of the fold induction of the TFBS-directed UR expression after treatment with the inducer of interest. TFBS marked with in red represent treatment-dependent effects on the TF library. The SEM values are calculated as 1 standard error of the mean each way.

|  | 1. **Microarray analysis** | | | 1. **qPCR analysis** | | |
| --- | --- | --- | --- | --- | --- | --- |
|  | TFBS | Log2 Fold Induction | *SEM* | TFBS | Log2 Fold Induction | *SEM* |
| **1** | **MRE** | **2.79** | ***0.35*** | **MRE** | **4.71** | ***0.28*** |
| **2** | **ARE** | **1.36** | ***0.04*** | **ARE** | **2.90** | ***0.53*** |
| 3 | CREB | 0.51 | *0.22* | NFAT | 0.71 | *0.31* |
| 4 | PXRE (ER6) | 0.39 | *0.24* | CHOP | 0.61 | *0.41* |
| 5 | AP1/TRE | 0.36 | *0.18* | PXRE (ER6) | 0.40 | *0.42* |
| 6 | RARE | 0.31 | *0.51* | YY1 (Ind) | 0.36 | *0.30* |
| 7 | YY1 (Ind) | 0.29 | *0.16* | Fra-1 | 0.26 | *0.59* |
| 8 | EGR | 0.23 | *0.16* | Myc | 0.24 | *0.33* |
| 9 | Oct | 0.20 | *0.21* | E2F/E2F-1 | 0.23 | *0.34* |
| 10 | E2F/E2F-1 | 0.17 | *0.11* | NRF1 | 0.21 | *0.38* |
| 11 | MEF-2A | 0.15 | *0.25* | c-Ets-2 | 0.21 | *0.16* |
| 12 | GAS | 0.14 | *0.21* | c-Jun | 0.21 | *0.16* |
| 13 | TCF/β-cat | 0.14 | *0.19* | Myb | 0.21 | *0.16* |
| 14 | AP2 | 0.13 | *0.35* | SOX | 0.20 | *0.68* |
| 15 | YY1 (Rep) | 0.13 | *0.13* | LEF-1 | 0.13 | *0.11* |
| 16 | Sp1 | 0.12 | *0.34* | Oct | 0.13 | *0.65* |
| 17 | STAT1 | 0.07 | *0.23* | TCF/β-cat | 0.11 | *0.35* |
| 18 | PBREM | 0.06 | *0.03* | CREB | 0.09 | *0.40* |
| 19 | ERE (Estrogen R) | 0.06 | *0.03* | GATA | 0.09 | *0.37* |
| 20 | NF-kB | 0.05 | *0.26* | Pax | 0.08 | *0.45* |
| 21 | ATF/ATF-1/ATF-2/ATF-3 | 0.02 | *0.70* | C/EBPα&β | 0.07 | *0.49* |
| 22 | c-Ets-2 | 0.00 | *0.30* | HSE | 0.06 | *0.37* |
| 23 | c-Jun | 0.00 | *0.20* | Ets | 0.04 | *0.42* |
| 24 | Myb | 0.00 | *0.20* | STAT | -0.03 | *0.10* |
| 25 | Xbp1 | 0.00 | *0.20* | Elk-1 | -0.04 | *0.57* |
| 26 | AP4 | 0.00 | *0.20* | FoxO | -0.05 | *0.17* |
| 27 | FoxO | -0.01 | *0.25* | Smad4 | -0.06 | *0.32* |
| 28 | c-Rel; RelA | -0.01 | *0.25* | LXRE | -0.06 | *0.89* |
| 29 | Lef/TCF | -0.06 | *0.05* | RARE | -0.08 | *0.34* |
| 30 | TARE | -0.06 | *0.40* | Egr-1 | -0.09 | *0.86* |
| 31 | C/EBPα&β | -0.08 | *0.12* | AP1/TRE | -0.10 | *0.62* |
| 32 | TGFβ | -0.10 | *0.12* | NF-kB | -0.11 | *0.53* |
| 33 | HIF1α | -0.11 | *0.52* | EGRE | -0.12 | *0.40* |
| 34 | LEF-1 | -0.11 | *0.06* | PBREM | -0.13 | *0.36* |
| 35 | Smad4 | -0.15 | *0.06* | Sp1 | -0.15 | *0.68* |
| 36 | CHOP | -0.15 | *0.16* | AP2 | -0.16 | *0.37* |
| 37 | AhRE | -0.25 | *0.50* | c-Rel; RelA | -0.16 | *0.60* |
| 38 | c-Ets-1 | -0.27 | *0.06* | P53 | -0.17 | *0.56* |
| 39 | STAT3 | -0.31 | *0.26* | GAS | -0.19 | *0.33* |
| 40 | GRE | -0.41 | *0.41* | TGFβ | -0.21 | *0.43* |
| 41 | EGRE | -0.41 | *0.06* | FoxA | -0.24 | *0.74* |
| 42 | Sp1 | -0.42 | *0.08* | ERE (Estrogen R) | -0.25 | *0.31* |
| 43 | STAT | -0.43 | *0.12* | TARE | -0.29 | *0.51* |
| 44 | Egr-1 | -0.48 | *0.29* | STAT | -0.32 | *0.40* |
| 45 | FoxA | -0.51 | *0.07* | STAT1 | -0.34 | *0.32* |
| 46 | STAT | -0.53 | *0.18* | MEF-2A | -0.34 | *0.59* |
| 47 | Pax | -0.53 | *0.48* | Lef/TCF | -0.36 | *0.28* |
| 48 | c-Fos | -0.60 | *0.34* | GRE | -0.38 | *0.42* |
| 49 | P53 | -0.63 | *0.17* | STAT3 | -0.38 | *0.19* |
| 50 | NFkB | -0.63 | *0.23* | HIF1α | -0.38 | *0.70* |
| 51 | SOX | -0.64 | *0.13* | PPRE | -0.45 | *0.67* |
| 52 | Myc | -0.67 | *0.35* | AP3 | -0.47 | *0.40* |
| 53 | PPRE | -0.67 | *0.27* | Sp1 | -0.50 | *0.44* |
| 54 | Elk-1 | -0.68 | *0.11* | c-Ets-1 | -0.52 | *0.28* |
| 55 | SRE; SRF | -0.69 | *0.46* | EGR | -0.56 | *0.80* |
| 56 | GRE (PRE, ARE, MRE) | -0.69 | *0.05* | c-Fos | -0.56 | *0.72* |
| 57 | GLI | -0.72 | *0.15* | AhRE | -0.58 | *0.66* |
| 58 | GATA | -0.72 | *0.05* | YY1 (Rep) | -0.60 | *0.38* |
| 59 | Fra-1 | -0.90 | *0.85* | ATF/ATF-1/ATF-2/ATF-3 | -0.64 | *0.51* |
| 60 | Ets | -0.95 | *0.16* | SRE; SRF | -0.68 | *0.22* |
| 61 | AP3 | -1.01 | *0.21* | GLI | -0.70 | *0.68* |
| 62 | LXRE | -1.03 | *0.34* | GRE (PRE, ARE, MRE) | -0.76 | *1.10* |
| 63 | NRF1 | -1.15 | *0.26* | ISRE | -0.92 | *0.43* |
| 64 | NFAT | -1.16 | *0.36* | AP4 | -1.15 | *0.42* |
| 65 | ISRE | -1.31 | *0.18* | NFkB | -1.33 | *0.47* |
| 66 | HSE | -1.35 | *0.36* | Xbp1 | -1.35 | *0.58* |
