# Supplementary material for: Unique Reporter-Based Sensor Platforms to Monitor Signalling in Cells
Source: PLoS One. 2012 Nov 29;7(11):e50521. doi: 10.1371/journal.pone.0050521 (PMC3510088; doi:10.1371/journal.pone.0050521)
Supplement: Table S4 — Changes in the levels of cAMP and cGMP in HEK293 cells treated with cyclic nucleotide analogues or PDEI. HEK293 cells transfected with pool of plasmids (listed in Table S2 and pRL-SV40) and were subsequently treated with chemicals of interest. Levels of intracellular cAMP and cGMP were quantified and are presented as the increase in the intracellular cAMP or cGMP levels in cells treated with inducers of interest compared to the levels in untreated cells. The errors are calculated as 1 standard error of the mean each way. Abbreviations: IBMX: 3-isobutyl-1-methylxanthine, EHNA: erythro-9-(2-hydroxy-3-nonyl)adenine. (DOCX) [file pone.0050521.s005.docx]

**Table S4: Changes in the levels of cAMP and cGMP in HEK293 cells treated with cyclic nucleotide analogues or PDEI.** HEK293 cells transfected with pool of plasmids (listed in Supporting Table 2 and pRL-SV40) and were subsequently treated with chemicals of interest. Levels of intracellular cAMP and cGMP were quantified and are presented as the increase in the intracellular cAMP or cGMP levels in cells treated with inducers of interest compared to the levels in untreated cells. The errors are calculated as 1 standard error of the mean each way. Abbreviations: IBMX: 3-isobutyl-1-methylxanthine, EHNA: erythro-9-(2-hydroxy-3-nonyl)adenine.

|  | PDEI | Selectivity | Substrates | cAMP | cGMP |
| --- | --- | --- | --- | --- | --- |
| HEK293 | - |  |  | 1 ± 0 | 1 ± 0 |
|  | + Forskolin |  |  | 14.09 ± 1.16 | nd |
|  | + 8-bromo-cAMP |  |  | 14.33 ± 2.20 | nd |
|  | + IBMX | non-specific | cAMP, cGMP | 19.19 ± 3.1 | 12.72 ± 0.89 |
|  | + EHNA | PDE2 | cAMP, cGMP | 14.72 ± 1.68 | 13.32 ± 0.56 |
|  | + Rolipram | PDE4 | cAMP | 11.06 ± 0.73 | nd |
|  | + 8-bromo-cGMP |  |  | nd | 11.74 ± 1.97 |
|  | + Vardenafil | PDE5 | cGMP | nd | 14.24 ± 0.70 |
|  | + Sildenafil | PDE5 | cGMP | nd | 13.42 ± 0.85 |
